# A homozygous *loss‐of‐function* mutation in *PDE2A* associated to early‐onset hereditary chorea

**DOI:** 10.1002/mds.27286

**Published:** 2018-02-02

**Authors:** Vincenzo Salpietro, Belen Perez‐Dueñas, Kosuke Nakashima, Victoria San Antonio‐Arce, Andreea Manole, Stephanie Efthymiou, Jana Vandrovcova, Conceicao Bettencourt, Niccolò E. Mencacci, Christine Klein, Michy P. Kelly, Ceri H. Davies, Haruhide Kimura, Alfons Macaya, Henry Houlden

**Affiliations:** ^1^ Department of Molecular Neuroscience University College of London London United Kingdom; ^2^ Department of Pediatric Neurology Hospital Universitari Sant Joan de Déu Barcelona Spain; ^3^ CNS Drug Discovery Unit, Pharmaceutical Research Division Takeda Pharmaceutical Company Limited Fujisawa Japan; ^4^ Unit of Epilepsy, Sleep and Neurophysiology Hospital Universitari Sant Joan de Déu Barcelona Spain; ^5^ Center for Genetic Medicine Feinberg school of medicine, Northwestern University Chicago Illinois USA; ^6^ Institute of Neurogenetics University of Lübeck Lübeck Germany; ^7^ Department of Pharmacology, Physiology and Neuroscience School of Medicine, University of South Carolina Columbia South Carolina USA; ^8^ Department of Pediatric Neurology University Hospital Vall d'Hebron Barcelona Spain

**Keywords:** phosphodiesterase, striatum, chorea, movement disorders, PDE2A

## Abstract

**Background:** We investigated a family that presented with an infantile‐onset chorea‐predominant movement disorder, negative for *NKX2‐1, ADCY5*, and *PDE10A* mutations. **Methods:** Phenotypic characterization and trio whole‐exome sequencing was carried out in the family. **Results:** We identified a homozygous mutation affecting the GAF‐B domain of the 3’,5’‐cyclic nucleotide phosphodiesterase *PDE2A* gene (c.1439A>G; p.Asp480Gly) as the candidate novel genetic cause of chorea in the proband. PDE2A hydrolyzes cyclic adenosine/guanosine monophosphate and is highly expressed in striatal medium spiny neurons. We functionally characterized the p.Asp480Gly mutation and found that it severely decreases the enzymatic activity of PDE2A. In addition, we showed equivalent expression in human and mouse striatum of *PDE2A* and its homolog gene, *PDE10A*. **Conclusions:** We identified a *loss‐of‐function* homozygous mutation in *PDE2A* associated to early‐onset chorea. Our findings possibly strengthen the role of cyclic adenosine monophosphate and cyclic guanosine monophosphate metabolism in striatal medium spiny neurons as a crucial pathophysiological mechanism in hyperkinetic movement disorders. © 2018 The Authors. Movement Disorders published by Wiley Periodicals, Inc. on behalf of International Parkinson and Movement Disorder Society.

Chorea is a hyperkinetic movement disorder characterized by an excess of brief, continuous, unpatterned involuntary movements.^1^ Focal lesions of the striatum, degeneration, or functional dysregulation of medium spiny neurons (MSNs) that constitute ∼95% of the striatal cells are considered to be crucially implicated in the pathophysiology of choreic movements.^2^, ^3^, ^4^, ^5^ A variety of acquired causes may underlie chorea in the pediatric age group (e.g., Sydenham chorea, cerebral palsy), but genetic etiologies also play a role in different early‐onset choreic syndromes. Among the possible genetic causes, dominantly inherited (or de novo) mutations in *NKX2‐1* (MIM #600635), encoding a transcription factor essential for striatum development, cause a variable spectrum of childhood‐onset disorders ranging from choreoathetosis to myoclonus, congenital hypothyroidism, and respiratory distress.^6^ In addition, de novo or dominantly inherited mutations in *ADCY5* (MIM #600293), encoding an enzyme crucial to the synthesis of cyclic adenosine monophosphate (cAMP) in MSNs, have been linked to different infantile/childhood‐onset phenotypes defined as “*ADCY5*‐related dyskinesias” that include nonprogressive choreiform movement disorders as well as pleiotropic fluctuating/paroxysmal dyskinesias.^7^, ^8^ Furthermore, both de novo dominant and biallelic mutations in the *PDE10A* gene (MIM #610652), encoding an enzyme involved in the hydrolysis/degradation of cAMP and cyclic guanosine monophosphate (cGMP) in MSNs, have been reported in patients with infantile/childhood‐onset chorea usually exhibiting a generalized distribution.^9^, ^10^ In this study, we performed trio‐based whole‐exome sequencing (WES) in a 12‐year‐old patient presenting a childhood‐onset chorea‐predominant movement disorder and identified a homozygous missense mutation (c.1439A>G; p.Asp480Gly) in *PDE2A* (MIM #602658), a gene mainly involved in brain cAMP and cGMP metabolism and not previously associated with human phenotypes, as the possible novel genetic cause of chorea in the patient. Screening of genomic data from a number (n = 62) of similarly affected individuals did not identify additional *PDE2A* likely pathogenic variants.

## Materials and Methods

### Genetic Analysis

**Figure 1:**
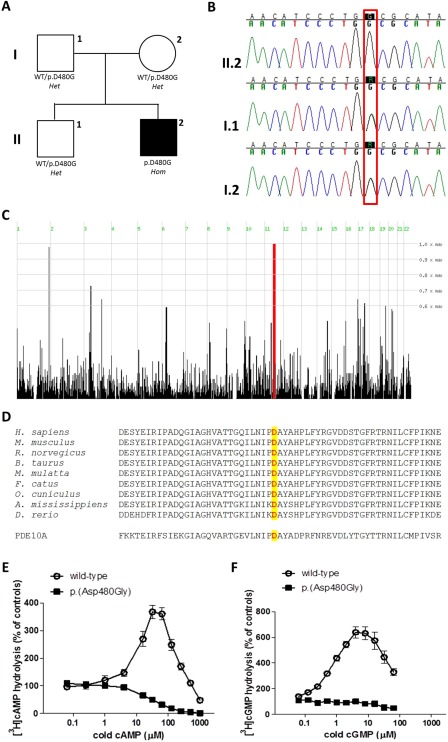
Family tree and genetic and functional studies. (A) Family tree. (B) Chromatograms from Sanger sequencing from individuals I‐1, I‐2, and II‐2. (C) Genetic analysis showing the homozygous block of the proband on chromosome 11 (chr11: 63138482‐76853783). (D) Multiple‐sequence alignment showing conservation of protein sequence across species and PDE homolog (PDE10A) in the GAF‐B domain, in which the p.Asp480Gly homozygous mutation (underlined) was found. (E) Catalytic activity of wild‐type and mutant PDE2A in the presence of 70 nM of [^3^H]cAMP. (F) Catalytic activity of wild‐type and mutant PDE2A in the presence of 70 nM of [^3^H]cGMP. [Color figure can be viewed at wileyonlinelibrary.com]

**Figure 2:**
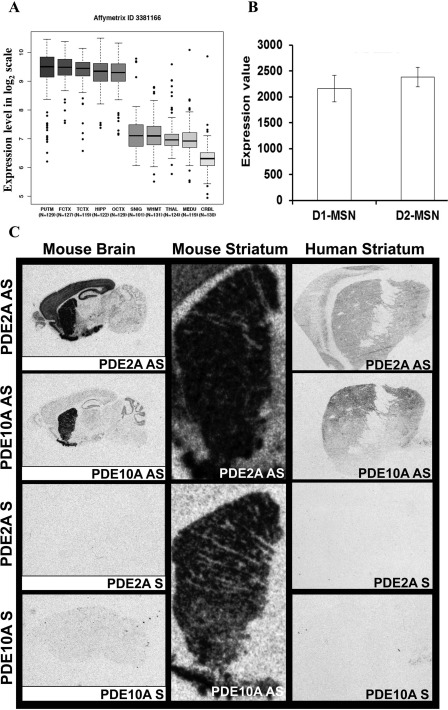
*PDE2A* expression studies. (A) Brain expression values of *PDE2A* show higher expression levels in the striatum and also other CNS regions, especially FCTX (frontal cortex) and TCTX (temporal cortex), and less in HIPP (hippocampus), OCTX (occipital cortex), WHMT (white matter), SNIG (substantia nigra), MEDU (medulla), THAL (thalamus), and CRBL (cerebellar cortex). (B) Expression values of *PDE2A* mRNA (1447707_s_at) in MSNs of direct and indirect pathways (D1 + and D2 + MSNs). (C) mRNA expression analysis on mice and human brains of *PDE2A* and *PDE10A* show similar expression patterns in striatum, but different expression patterns elsewhere in mouse and human brain.

The 12‐year‐old male patient is the second child of unrelated healthy parents, both originally from the Canary Island of Tenerife, and he was seen at the Hospital Sant Joan de Déu in Barcelona (Spain) by movement disorder specialists because of a history of early‐onset fluctuating dyskinesia associated with chronic chorea. To molecularly investigate the cause of the disease in this child, the 4 family members (Fig. 1A) donated their blood samples after informed consent and DNA was extracted using standard procedures. A trio WES study of the family (Fig. 1A, I‐1, I‐2, II‐2) was then performed. Genomic pipeline and variants annotation were carried out as previously reported^11^, ^12^ and described in the Supplementary Information. In accord with the pedigree and phenotype, our filtering strategy prioritized rare (<1% in public databases, including 1000 Genomes project and Exome Aggregation Consortium [ExAC v0.2]) variants that were fitting a de novo or a recessive model (Supplementary Information). We also performed homozygosity mapping in the family (Supplementary Information).

### Functional Characterization of the p.Asp480Gly *PDE2A* Mutation

#### Materials

cAMP and cGMP were purchased from Sigma‐Aldrich (St. Louis, MO). The [^3^H]‐labeled nucleotides, [^3^H]cAMP (31.3 Ci/mmol) and [^3^H]cGMP (14.3 Ci/mmol), were purchased from PerkinElmer (Waltham, MA).

#### Cloning and Expression of Constructs

Complementary DNA for human PDE2A3 (GenBank: U67733) was used as a template and the mutant, c.1439A>G; p.(Asp480Gly), was constructed by site‐directed point mutation. All constructs were cloned into the pcDNA3.1(+)neo vector (Thermo Fisher Scientific, Inc., Waltham, MA) and transfected into COS‐7 cells (ECACC, Salisbury, UK). The membrane fractions were used for the enzyme assay.

#### In Vitro Phosphodiesterase Enzyme Assay

Phosphodiesterase (PDE) activities were measured using a scintillation proximity assay (SPA)‐based method.^13^ In this assay, the product of the PDE reaction, either [^3^H]AMP or [^3^H]GMP, can bind directly to yttrium silicate PDE SPA beads (GE Healthcare Ltd., Little Chalfont, UK), leading to light emission from the scintillant in the beads. The enzyme assays were conducted in a buffer (50 mM of HEPES‐NaOH, 8.3 mM of MgCl_2_, 1.7 mM of ethylene glycol tetraacetic acid, and 0.1% bovine serum albumin [pH 7.4]) in 96‐well half‐area plates (Corning Inc., Corning, NY). For enzyme studies, reaction was conducted at the presence of the indicated concentrations of substrate using a mixture (20 µL) of [^3^H]‐labeled and unlabeled cAMP or [^3^H]‐labeled and unlabeled cGMP with the 20 µL of membrane fractions of PDE2A‐expressing COS‐7 cells at 37^o^C, followed by reaction termination by SPA beads addition (20 µL of 20 mg/mL). Degradation of each radiolabeled substrate ([^3^H]cAMP or [^3^H]cGMP) at the presence of various concentrations of cold cAMP or cGMP, respectively, was measured using the SPA‐based assay method.

### PDE2A Messenger RNA Expression Studies

Overall brain expression and cell‐specific expression data were initially obtained using BRAINEAC and BacTRAP mice data, respectively.^14^, ^15^ Then, we analyzed in vivo *PDE2A* and *PDE10A* messenger RNA (mRNA) expression patterns using mice and human brains, as described in detail in the Supplementary Information.

## Results

Disease onset in our patient mainly consisted in fluctuating attacks of sudden falls, followed by dystonic postures and generalized choreic movements. Subsequently, he developed since the age of 9 years a slowly progressive choreic movement disorder associated with dystonic features. In addition to his movement disorder, he also had language difficulties, cognitive impairment (total score 44 with Kauffman Brief Intelligence Test), and a history of interictal epileptic features (Supplementary Information). We performed a trio‐based WES, and, after applying our filtering criteria, we identified a number of possibly pathogenic variants according to guidelines for variants interpretation (Supplementary Information; Supplementary Table 1).^16^ The proband of our family carried a single de novo variant in the gene, *STRADA* (NM_153335.5; c.1042G>T: p.Gly348Trp); homozygous intragenic deletions (or biallelic truncating mutations) in this gene were previously associated with polyhydramnios, megalencephaly, distinctive facial features, and symptomatic epilepsy (#MIM 611087),^17^, ^18^ a phenotype not consistent with the clinical presentation of our patient. In addition, we identified a homozygous variant (NM_002599.4; c.1439A>G: p.Asp480Gly) in *PDE2A* (#MIM 602658) as the only biallelic variant segregating with the disease within the family. Also, the *PDE2A* homozygous mutation was inherited within the only significant homozygous block (chr11: 63138482‐76853783) identified in the proband by homozygosity mapping analysis (Fig. 1B–D; Supplementary Information). Additionally, our in vitro functional studies showed that both cAMP and cGMP hydrolysis were markedly increased for wild‐type PDE2A compared to the p.Asp480Gly mutant (approximately 3.7‐fold and 6.4‐fold, respectively). On the contrary, neither cAMP nor cGMP increased enzyme activities of the mutant enzyme (Fig. 1E,F; Supplementary Tables 2‐5), indicating a severe disruption of catalytic activity of mutated PDE2A.

## Discussion

The genetic analysis of this family using WES and homozygosity mapping indicated a homozygous missense mutation in *PDE2A* as the most likely explanation for the patient's disease. This is supported by: (1) in silico pathogenic predictors, conservation, and cosegregation analysis (Supplementary Table 1; Fig. 1D); (2) previous^9^, ^10^, ^19^ and current (Fig. 2A–C) studies, which indicates a high (predominant) expression of *PDE2A* in the striatum (qualitatively equivalent to *PDE10A* striatal expression); (3) biological importance of the PDE2A GAF‐B domain (where the p.Asp480Gly mutation is located) in the metabolism of cAMP and cGMP (and implication of these cyclic nucleotides in the pathogenesis of similar phenotypes including *ADCY5*‐ and *PDE10A*‐related movement disorders)^5^, ^6^, ^7^, ^8^, ^9^, ^10^, ^20^, ^21^, ^22^, ^23^; and (4) in vitro functional studies that showed a severe disruption of the mutant PDE2A enzymatic activity (Fig. 2E; Supplementary Tables 2‐5).

Importantly, the amino acid involved by the mutation we identified (aspartic acid at position 480) is located in the presumed cyclic nucleotide binding pocket of GAF‐b domain.^24^ To assess enzyme activity of PDE2A, we measured degradation of radiolabeled substrate ([^3^H]cAMP or [^3^H]cGMP) and showed a significant decrease in the hydrolytic activity of the mutant PDE2A enzyme. Of note, we screened genomic data from 17 individuals with childhood‐onset chorea and from 45 patients presenting fluctuating or paroxysmal dyskinesia and failed to identify further mutations in *PDE2A*, suggesting that this might represent a very rare genetic cause of early‐onset choreic/hyperkinetic movement disorders. The *PDE2A* p.Asp480Gly mutation was inherited within the only homozygous block on a shared haplotype (Supplementary Fig. 1), indicating that this could possibly represent a founder mutation from the genetic isolate of Canary Islands. Supporting this Canary founder effect, in the ExAC database (http://exac.broadinstitute.org, last accessed October 2017) containing 60,706 individuals, the *PDE2A* gene was found to be highly constrained for missense variation (z = 4.78), with only 27 individuals carrying nonsynonymous (heterozygous) variants affecting the GAF‐B domain.

Of interest, monogenic etiologies underlying infantile‐ and childhood‐onset choreic movement disorders are being increasingly recognized.^7^, ^8^, ^9^, ^10^ In this regard, the identification and characterization of the *ADCY5*‐ and *PDE10A*‐related spectrum of disorders shed new light on the key role of cAMP and cGMP signaling in basal ganglia circuit and the control of movements.^5^, ^6^, ^7^ The adenyl cyclase 5 enzyme catalyzes cAMP formation and pathogenic *ADCY5* mutations might thus increase the synthesis of cAMP because of a possible enhancement of Ac5 enzymatic activity.^5^, ^7^, ^8^ The PDE10A enzyme is involved in cAMP hydrolysis in MSNs, and in vitro assessment of *PDE10A* mutations showed that both dominant and recessive variants lead to a *loss‐of‐function* effect with consequent impairment of cAMP and cGMP hydrolysis/degradation.^9^, ^10^ Thus, elevated cAMP (and possibly cGMP) intracellular levels in MSNs (either attributed to increased synthesis or reduced hydrolysis) could represent a central mechanism for molecular pathogenesis of different hyperkinetic movement disorders.^5^ Notably, the cyclic nucleotides cAMP and cGMP are ubiquitous intracellular second messengers regulating a variety of biological processes.^25^ The intracellular concentration of these molecules is modulated by the activity of PDEs, a class of several different enzymes currently grouped overall in 11 families, and, among the PDEs, *PDE2A* and *PDE10A* are among the most highly enriched in the striatum.^26^, ^27^ *PDE2A* expression is highest in the striatum, cortex, and hippocampus compared to other central nervous system (CNS) regions (Fig. 2A), with similar expression in direct and indirect MSN pathways (Fig. 2B). Furthermore, our comparative expression analysis on human and mice brain found that striatal mRNA levels of *PDE2A* and its paralogue gene, *PDE10A*, are qualitatively equivalent, with a more generalized *PDE2A* expression in some additional CNS regions (Fig. 2C). These *PDE2A* more generalized brain expression patterns may explain the broad neurological features (including interictal epileptic discharges and cognitive impairment) we observed in our patient in addition to his movement disorder. Notably, the PDE2A enzyme is a dual‐substrate PDE that hydrolyzes both cAMP and cGMP and has two N‐terminal tandem noncatalytic domains, named GAF‐A and GAFB.^18^, ^19^, ^28^ PDE2A exhibits a low level of basal hydrolytic activity that is further stimulated when its GAF‐B domain binds cAMP or cGMP.^20^, ^21^, ^29^ However, it is most likely that only cGMP binds the PDE2A GAF‐B domain in vivo, thereby selectively stimulating its cAMP hydrolytic activity.^20^, ^30^

In conclusion, results from our genetic and functional studies indicate a *PDE2A* p.Asp480Gly homozygous *loss‐of‐function* mutation as the likely genetic cause of early‐onset hereditary chorea in our family, thus possibly expanding the genetic aetiology of early‐onset choreic/hyperkinetic movement disorders associated to abnormal c‐AMP and c‐GMP metabolism in striatal MSNs.

## Legend to the Video

The episodes of dyskinesia initiated with neck extension, backward falling, and dystonic posturing of the four limbs, followed by choreic movements, facial grimacing, blinking, and orolingual movements. During execution of motor task (writing), the child showed a complex hyperkinetic movement consisting in baseline chorea associated with dystonic posturing (predominantly in the left foot).

## Author Roles

(1) Research Project: A. Conception, B. Organization, C. Execution; (2) Statistical Analysis: A. Design, B. Execution, C. Review and Critique; (3) Manuscript Preparation: A. Writing of the First Draft, B. Review and Critique.

V.S.: 3A, 3B

B.P.‐D.: 1A, 1B, 1C, 3A

K.N.: 1A, 1B, 1C, 3A

V.S.A.‐A.: 1A, 1B, 1C, 3A

A. Manole: 3B

S.E.: 1A, 1B, 1C, 3A

J.V.: 1B, 1C, 3A

C.B.: 1A, 1B, 1C, 3A

N.E.M.: 1A, 1B, 1C, 3A

C.K.: 3B

M.P.K.: 1A, 1B, 1C, 3A

C.H.D.: 3B

H.K.: 3B

A. Macaya: 3B

H.H.: 3B

## Financial Disclosures

Nothing to report.

## Supporting information

Additional Supporting Information may be found in the online version of this article at the publisher's website

Supplementary Information

**Supplementary FIG. 1**. Haplotype analysis for the region on chromosome 11 surrounding *PDE2A* c.1439A>G (indicated in red), with markers and their positions (Bp) displayed on the left.

Supplementary Information Tables

Supplementary Information Movie
