# Supplementary material for: A homozygous loss‐of‐function mutation in PDE2A associated to early‐onset hereditary chorea
Source: Mov Disord. 2018 Feb 2;33(3):482–8. doi: 10.1002/mds.27286 (PMC5873427; doi:10.1002/mds.27286)
Supplement: Supplementary file 1 — Supplementary Information [file MDS-33-482-s001.docx]

**Supplementary Section**

**Phenotypic characterization**

The 12-year-old male patient is the second child of unrelated healthy parents, both originally from the Canary Island of Tenerife, and he was seen and followed-up at the Hospital Sant Joan de Déu in Barcelona, Spain. Family history revealed no history of movement disorders. He had a healthy 15-year-old brother.

He was born at term (41 weeks of gestational age), following an uneventful pregnancy and delivery. At birth growth parameters and head circumference were within normal ranges. First concerns appeared in early development, with language delay, poor coordination and low academic abilities. Follow-up psychometric tests revealed moderate intellectual disability with static scores. Last evaluation at the age of 11 years disclosed low scores in cognitive Kauffman Brief Intelligence Test (total score 44) and Vinelland II adaptive behaviour scale (total score 59) (mean 100 ± 15 standard deviation). He had a convulsive seizure in febrile context at age 2 years. At this age, he also presented with frequent episodes consisting of sudden falls, followed by generalized choreic movements and dystonic postures. They occurred several times a day (usually lasting a few seconds) and were triggered by emotional stress, sudden movements (e.g., getting up from a chair or getting into the car), or during motor planning. The patient did not lose consciousness during these episodes.

A 24 hours video-electroencephalographic (VEEG) monitoring at the age of 5 years old identified up to 100 episodes as previously described, most of them with the patient being awake and only very few episodes recorded during sleep. The episodes were initiated with neck extension, backward falling and dystonic posturing of the four limbs, followed by choreic movements, facial grimacing, blinking, and orolingual movements (Supplementary Video). There was no clear EEG ictal pattern during the episodes. Background activity was poorly organized. There were frequent bilateral asynchronous centro-parietal and fronto-temporal interictal epileptic discharges, more common during sleep. Brain magnetic resonance imaging (MRI) was normal. Ictal SPECT did not localize any region of seizure onset. 18F-fluorodeoxyglucose (18F-FDG) Positron Emission Tomography showed normal uptake in all brain areas. Metabolic investigations in cerebrospinal fluid (CSF) revealed normal CSF/blood glucose ratio, biogenic amines and folate. Genetic studies performed (which included *NKX2-1*, *PDE10A* and *ADCY5* Sanger sequencing and a comparative genome hybridization analysis using qChip post array) were all normal.

The patient was diagnosed with epilepsy with possibly focal onset seizures. Several trials with antiepileptic drugs (valproate, levetiracetam, oxcarbazepine, carbamazepine, perampanel, lamotrigine, phenobarbital, rufinamide, zonisamide, flunarizine, topiramate and clobazam) were ineffective. Vagus nerve stimulation (VNS) therapy at the age of 6 years did not reduce the falls.

Since the age of 9 years the child developed a slowly progressive movement disorder, consisting in baseline choreic movements associated with dystonic posturing predominantly in the left foot, that fluctuated according to the frequency of attacks (Supplementary Video).

At the age of 11 years, according to the clinical evolution of the movement disorder, the clinical phenomenology of the attacks, and the lack of ictal EEG abnormalities on a repeated VEEG monitoring, we established the diagnosis of fluctuating dyskinesia associated with chronic chorea. We decided to withdrawal antiepileptic drugs with the exception of carbamazepine, maintained due to the active interictal epileptic abnormalities, and performed bilateral GPi stimulation.

Globus pallidus internus (GPi) electrodes and the neurostimulator Activa RC (Medtronic®) were implanted in the same surgical session. Electrode insertion was assisted by the robotic arm Neuromate® (Renishaw®). Four months after deep brain stimulation the baseline choreic movements have improved, and the patient has regained independence in his daily living activities. Fluctuating dyskinetic attacks have been reduced in frequency (from up to one hundred to less than fifty episodes per day) and intensity (they rarely produce falls on walking).

**Genetic analysis**

To investigate the genetic cause of the disease in the patient, trio whole-exome sequencing (WES) was performed in the proband (Figure 1A: II-2) and his healthy parents (Fig.1: I-1 and I-2). Nextera Rapid Capture Enrichment kit (Illumina) was used according to the manufacturer instructions. Libraries were sequenced in an Illumina HiSeq3000 using a 100-bp paired-end reads protocol. Sequence alignment to the human reference genome (UCSC hg19), and variants call and annotation were performed using an in-house pipeline as described elsewhere [11, 12]. WES generated a total of 71,369,726 (Proband, Fig. 1A: II-1), 74,615,568 (Father, Fig. 1A: I-1) and 83,817,668 (Mother, Fig. 1A: I-1) unique reads, with an average on target depth over 150, and >98% of the target bases covered at least 10X. The raw list of single nucleotide variants (SNVs) and indels was filtered. Only exonic and donor/acceptor splicing variants were considered. In accordance with the pedigree and phenotype, priority was given to rare [<1% in public databases, including 1000 Genomes project, NHLBI Exome Variant Server, Complete Genomics 69, and Exome Aggregation Consortium (ExAC v0.2)] variants **(i)** fitting a recessive model (i.e., homozygous in the Proband but heterozygous in the parents or compound heterozygous in the Proband but not in the parents), **(ii)** *de-novo*, or **(iii)** located in genes previously associated with movement disorders or neurological phenotypes. After applying the above filtering criteria using the trio-based WES data, we identified in the Proband 1 *de-novo* variant (NM_153335.5; c.1042G>T: p.Gly348Trp) in the gene *STRADA,* 1 compound heterozygous variant in the gene *SH3TC1* (NM_018986.3; c.353G>A: p.Arg118Gln and c.2890C>T: p.Gln964*) and 1 homozygous variant (NM_002599.4; c.1439A>G: p.Asp480Gly) in *PDE2A* (Supplementary Table 1). These identified variants were all absent in our in-house exome database (containing over than 6,000 exomes from both healthy individuals and patients with heterogeneous neurological disorders) and were confirmed by traditional Sanger sequencing. Segregation analysis of the 2 biallelic variants identified the compound heterozygous mutations in *SH3TC1* (NM_018986.3; c.353G>A: p.Arg118Gln and c.2890C>T: p.Gln964*) also in the unaffected brother (Fig. 1A: II-1(, thus excluding co-segregation with the disease. The mutation in *PDE2A* (NM_002599.4; c.1439A>G: p.Asp480Gly) was heterozygous in the healthy parents and the unaffected brother and homozygous exclusively in the Proband (Fig. 1B). The *de-novo* variant (NM_153335.5; c.1042G>T: p.Gly348Trp) in *STRADA* affected a non conserved genomic position (GERP ++ score 0.7) and is absent in ExAC, although the amino acid involved in the substitution (glycine) is replaced in 6 individuals by either a glutamate (n=4) or a valine (n=2) residue (http://exac.broadinstitute.org, last accessed October 2017).

Patients carrying homozygous intragenic deletions or biallelic truncating point mutations in *STRADA* have been described with epilepsy associated to a number of additional features (polyhydramnios, megalencephaly, distinctive facial features) [17, 18], which were absent in our Patient. However, we performed a copy number variants analysis to evaluate possible intragenic deletions/insertions in *STRADA* in our Patient.  WES data were analysed using the two read depth-based software algorithms CoNIFER [31] and CANOES [32]. For both callers, samples (n=151) from the same sequencing batch (which included WES data of the Proband and his parents) were analysed simultaneously. No significant deviations in read depth were detected using either tool in the Proband in *STRADA*, making unlikely the presence of a copy number variant in the other allele.

In order to investigate the presence of homozygosity regions in the Proband inherited on the same haplotype we performed homozygosity mapping in the individuals from the family analysing WES VCF files on Homozygosity Mapper ([www.homozygositymapper.org](http://www.homozygositymapper.org)). We identified a single block of homozygosity consisting in a 13-M region that contains *PDE2A* (in which the p.Asp480Gly mutation was found) on chromosome 11 (chr11: 63138482-76853783). For the haplotype analysis, we removed single nucleotide polymorphisms which were in approximate linkage equilibrium with each other and a pruned subset of SNPs was generated using PLINK 1.9 from WES trio (VCF) data. Only SNPs on this list, belonging to chromosome 11 and genotyped for all three individuals in the family were kept, and haplotypes for chromosome 11 were then inferred using MERLIN 1.1.2 (Supplementary Figure 1).

***PDE2A* Expression studies**

The brain expression and the cell-specific expression data, were obtained using BRAINEAC and BacTRAP mice, respectively [14, 15] and downloaded from the Gene Expression Omnibus (GEO) database: <http://www.ncbi.nlm.nih.gov/geo/>. The used GEO accession numbers for the BacTRAP mice are GSM337807, GSM337808, and GSM337809 for the Drd1^+^ MSNs and GSM337812, GSM337813, GSM337814 for the Drd2^+^ MSNs. In addition to *in-silico* expression analysis we performed *in-vivo* analysis of *PDE2A* and *PDE10A* mRNA expression patterns in mice and human brain. Autoradiographic *in situ* hybridization was carried out generally as previously described. Rodents were killed by cervical dislocation without anaesthesia. Brains were flash frozen in isopentane sitting on dry ice and then stored at -80. Brains were then cryosectioned at 20 µM, and sections were thaw mounted on slides and then stored at -80°. Slides with 14 µM-thick sections of human striatum were obtained from the Stanley Foundation and stored at -80°. Upon thawing, slides were fixed in 4% paraformaldehyde for 10 minutes and then treated with 0.1 M Triethanolamine/0.9% NaCl for 10 minutes. Sections were then dehydrated through an ethanol series (50-80%-90%-100%, each 1 minute), defatted in chloroform (2 x 5 minutes), then rehydrated through an ethanol series ending in water (100%-100%-90%-80%-50%-H2O, each 1 minute). Slides were allowed to air dry and then placed in hybridization trays, the bottoms of which held 2X sodium citrate buffer (SSC) to create a moist environment. Antisense or sense (negative control) oligonucleotide probes specific to PDE2A (antisense: 5'- AAC TTG CTG AAC CAT GGC CCA TTG ATC TTG TTC AC -3') or PDE10A (antisense: 5'- CAT ATA TGC ATA TAT ACT GGC TGT TTG AAT TAT GAA TTT A -3') were labeled with S^35^-dATPs and then 600,000 cpms of probe was added per 100 µL of hybridization buffer (8.4% dextran sulfate/50% formamide/25mM HEPES/1mM EDTA/0.1M DTT/4% salmon sperm/4% poly rA/1X Denhardts solution/0.6M NaCl). 100 µL of labelled hybridization buffer was added to the rodent slides, and 400 µL of labelled hybridization buffer was added to the human slides. Each were covered with a glass coverslip and incubated overnight in a humid oven set to 32° C. The next day, coverslips were gently removed with 2X SSC and then slides were washed in 2X SSC (2 x 10 minutes room temperature), 0.2X SSC (2 x 1 hour at 50° C), and 2X SSC (1 x 10 minutes at room temperature) and then briefly rinsed in sterile water and then 70% EtOH. Slides were opposed to Kodak BioMax MR films for 2 months and images captured using a CoolSnap EZ digital camera and MCID software.
