# Supplementary material for: A homozygous loss‐of‐function mutation in PDE2A associated to early‐onset hereditary chorea
Source: Mov Disord. 2018 Feb 2;33(3):482–8. doi: 10.1002/mds.27286 (PMC5873427; doi:10.1002/mds.27286)
Supplement: Supplementary file 2 — Supplementary FIG. 1. Haplotype analysis for the region on chromosome 11 surrounding PDE2A c.1439A>G (indicated in red), with markers and their positions (Bp) displayed on the left. [file MDS-33-482-s002.pdf]

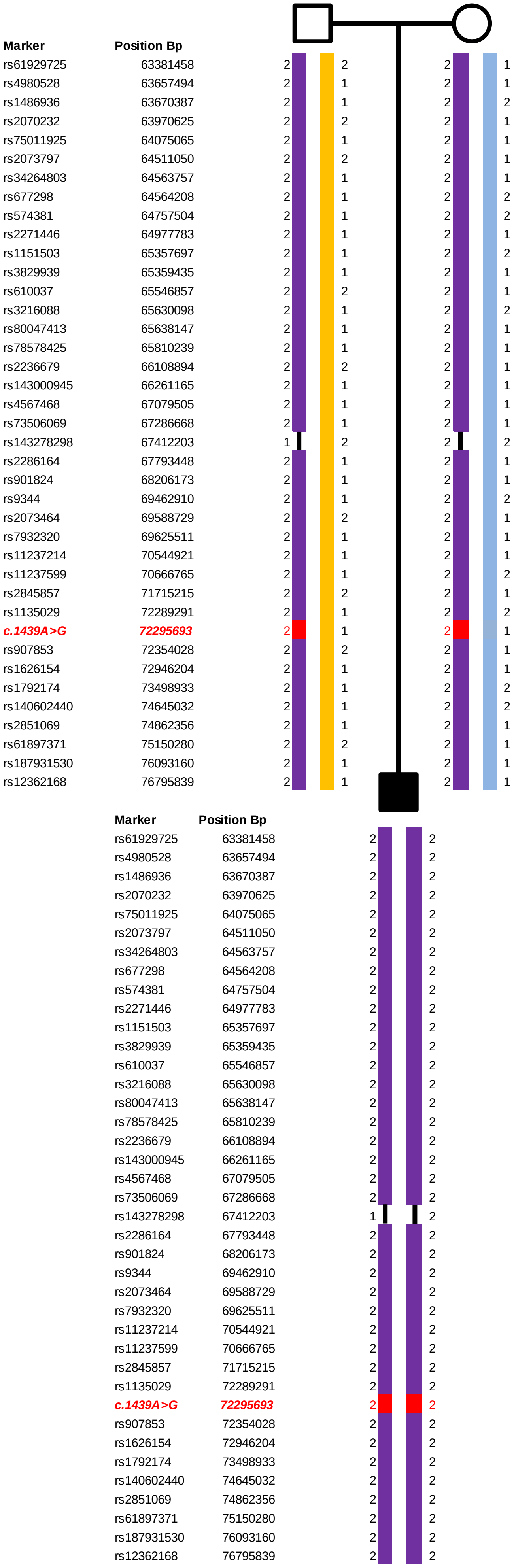

Supplementary Figure 1. Haplotype analysis for the region on chromosome 11 surrounding *PDE2A* c.1439A>G (indicated in red), with markers and their positions (Bp) displayed on the left.
