# Supplementary material for: A homozygous loss‐of‐function mutation in PDE2A associated to early‐onset hereditary chorea
Source: Mov Disord. 2018 Feb 2;33(3):482–8. doi: 10.1002/mds.27286 (PMC5873427; doi:10.1002/mds.27286)
Supplement: Supplementary file 3 — Supplementary Information Tables [file MDS-33-482-s003.docx]

**Supplementary Table 1. Filtered *de-novo* or biallelic variants in our Patient**

| **Gene** | **Inheritance** | **Transcript** | **Mutation** | **SIFT** | **PolyPhen-2** | **Mutation Taster** | **GERP^++^** | **ExAC** | **Uniprot informations** | **Variant interpretation (ACMG Guidelines) [16]** |
| --- | --- | --- | --- | --- | --- | --- | --- | --- | --- | --- |
| *STRADA* | *De-novo* | NM_153335.5 | c.1042G>T; p.Gly348Trp  (Heterozygous) | Deleterious | Deleterious | Pathogenic | 0.7 | Absent | Pseudokinase which, in complex with CAB39/MO25 (CAB39/MO25alpha or CAB39L/MO25 beta), binds to and activates STK11/LKB1. Adopts a closed conformation typical of active protein kinases and binds STK11/LKB1 as a pseudosubstrate, promoting conformational change of STK11/LKB1 in an active conformation | **∙** Missense variant in a gene for which primarily bi-allelic truncating variants are known to cause disease  **∙** Computational evidence suggest no impact on gene or gene product (low conservation score) |
| *SH3TC1* | Compound heterozygous | NM_018986.3 | c.353G>A: p.Arg118Gln;c.2890C>T: p.Gln964* (Compound heterozygous) | Not deleterious (Missense) | Not deleterious (Missense) | Not deleterious (Missense) | 2.55 (Missense) | 5.121e-05 (Missense) | SH3 domain and tetratricopeptide repeat-containing protein 1 | **∙** Lack of segregation (the compound heterozygous variants are present in an unaffected family member) |
| *PDE2A* | Autosomal Recessive | NM_002599.4 | c.1439A>G; p.Asp480Gly (Homozygous) | Deleterious | Deleterious | Pathogenic | 5.51 | Absent | Cyclic nucleotide phosphodiesterase with a dual-specificity for the second messengers cAMP and cGMP, which are key regulators of many important physiological processes. | **∙** Well-established in vitro functional studies supportive of a damaging effect on the gene product  **∙** Located in an important functional domain (GAF-B domain) without benign variation (in the biallelic state) in public databases  **∙** Co-segregation with the disease in the family  **∙** Multiple lines of computational (and empirical) evidence support a deleterious effect on the gene or gene product (conservation, evolutionary, implication of the paralog gene in similar phenotypes) |

**Supplementary Table 2. Wild-type Pde2a enzymatic activities for cAMP hydrolysis**

| cAMP (μM) | [^3^H] cAMP hydrolysis (% of controls) | | | |
| --- | --- | --- | --- | --- |
|  | N=3 | 1 | 2 | 3 |
| 1024 |  | 50.16 | 44.27 | 48.94 |
| 512 |  | 144.1 | 110.81 | 104.90 |
| 256 |  | 179.47 | 185.54 | 144.23 |
| 128 |  | 283.31 | 249.04 | 214.36 |
| 64 |  | 403.93 | 340.93 | 336.92 |
| 32 |  | 375.86 | 405.33 | 322.56 |
| 16 |  | 316.28 | 269.25 | 222.40 |
| 4 |  | 164.11 | 153.17 | 123.62 |
| 1 |  | 131.14 | 141.91 | 87.02 |
| 0.25 |  | 106.31 | 122.04 | 77.02 |
| 0.0625 |  | 112.44 | 90.42 | 85.83 |

**Supplementary Table 3. Mutant (p.Asp480Gly) Pde2a enzymatic activity for cAMP hydrolysis**

| cAMP (μM) | [^3^H] cAMP hydrolysis (% of controls) | | | |
| --- | --- | --- | --- | --- |
|  | N=3 | 1 | 2 | 3 |
| 1024 |  | 0.28 | 2.50 | -0.09 |
| 512 |  | 2.48 | 7.12 | 5.91 |
| 256 |  | 2.03 | 9.09 | 11.61 |
| 128 |  | 16.44 | 18.38 | 20.67 |
| 64 |  | 31.25 | 26.91 | 35.95 |
| 32 |  | 46.40 | 40.14 | 62.67 |
| 16 |  | 56.59 | 59.93 | 78.85 |
| 4 |  | 93.24 | 86.32 | 104.27 |
| 1 |  | 102.20 | 94.60 | 103.41 |
| 0.25 |  | 100.06 | 100.45 | 111.35 |
| 0.0625 |  | 94.14 | 114.69 | 118.26 |

**Supplementary Table 4. Wild-type Pde2a enzymatic activities for c-GMP hydrolysis**

| cGMP (μM) | [^3^H] cGMP hydrolysis (% of controls) | | | |
| --- | --- | --- | --- | --- |
|  | N=3 | 1 | 2 | 3 |
| 64 |  | 278.49 | 340.04 | 367.61 |
| 32 |  | 359.02 | 450.78 | 525.07 |
| 16 |  | 452.78 | 586.53 | 685.75 |
| 8 |  | 540.68 | 640.59 | 714.33 |
| 4 |  | 583.06 | 610.69 | 725.95 |
| 2 |  | 506.54 | 533.35 | 593.83 |
| 1 |  | 425.51 | 408.76 | 480.70 |
| 0.5 |  | 319.55 | 286.27 | 346.20 |
| 0.25 |  | 211.51 | 189.43 | 247.47 |
| 0.125 |  | 171.21 | 170.27 | 172.54 |
| 0.0625 |  | 133.11 | 137.43 | 145.92 |

**Supplementary Table 5. Mutant (p.Asp480Gly) Pde2a enzymatic activity for c-GMP hydrolysis**

| cGMP (μM) | [^3^H] cGMP hydrolysis (% of controls) | | | |
| --- | --- | --- | --- | --- |
|  | N=3 | 1 | 2 | 3 |
| 64 |  | 25.43 | 54.07 | 67.54 |
| 32 |  | 19.41 | 47.34 | 92.63 |
| 16 |  | 43.47 | 83.37 | 118.85 |
| 8 |  | 62.32 | 81.62 | 105.24 |
| 4 |  | 85.70 | 89.48 | 127.09 |
| 2 |  | 78.66 | 82.86 | 111.74 |
| 1 |  | 80.82 | 81.00 | 115.98 |
| 0.5 |  | 103.41 | 95.14 | 110.11 |
| 0.25 |  | 81.27 | 97.34 | 88.89 |
| 0.125 |  | 92.17 | 98.70 | 142.07 |
| 0.0625 |  | 90.69 | 90.89 | 148.44 |
